# Longitudinal Impact of the ACT-Based Positive Psychology Intervention to Improve Happiness, Mental Health, and Well-Being

**DOI:** 10.1007/s11126-025-10145-7

**Published:** 2025-04-22

**Authors:** Gökmen Arslan, Umut Aydoğdu, Kıvanç Uzun

**Affiliations:** 1 Department of Psychological Counseling and Guidance, Faculty of Education, Burdur Mehmet Akif Ersoy University, Burdur, Türkiye; 2 Centre for Wellbeing Science, University of Melbourne, Melbourne, Australia; 3 Department of Psychological Counseling and Guidance, Faculty of Education, Uşak University, Uşak, Türkiye

**Keywords:** Positive psychology, ACT, Well-being, Mental health, Intervention

## Abstract

This study aims to examine the effectiveness of ACT-based positive psychology intervention on young people's mental health and well-being. Existing literature offers limited insights into the impacts of ACT-based PPIs, particularly concerning young people. The study explores the potential of this intervention to enhance positive psychological outcomes such as subjective well-being, self-compassion, and optimism, while reducing negative psychological outcomes like pessimism, anxiety, depressive symptoms, and somatic symptoms, and improving resilience. Conducted at a state university, the study involved 42 participants between the ages of 19 and 23 who were assigned to experimental (*n* = 20) and control (*n* = 22) group. The experimental group participated in an 8-week ACT-based PPI program. Employing a longitudinal experimental design, the study included a pre-test, post-test, and a follow-up test approximately two years later. Results indicated that the experimental group experienced significant improvements in positive psychological outcomes and reductions in negative outcomes, with the effects sustained during the follow-up period. Notably, long-term enhancements in resilience and self-compassion were observed. Overall, this study provides important evidence of the effectiveness of ACT-based PPI in promoting young people's long-term outcomes, with implications for developing intervention programs to support their mental health and well-being.

## Introduction

One of the most important public health problems facing modern societies is the deterioration in the mental health and well-being of young people [18, 19]. Economic uncertainties, transformations in social relations and technological innovations have serious effects on the psychological health of young people in today's world where digitalization is gaining momentum [101]. While these issues are prevalent globally, local contexts present unique challenges. Young people are known to struggle with problems such as depression, anxiety disorders and loneliness, which negatively affect their functioning and quality of life [73]. According to previous studies and World Health Organization (WHO) data, depression rates are higher in young people compared to the general population, and it is estimated that 20% of individuals in this age group experience some form of mental health problem [61, 105]. Loneliness is another problem frequently reported by this group, and the lack of social relationships further undermines their mental health [9]. In addition, anxiety disorders and problems coping with stress are becoming increasingly common among individuals in this age group [26].

The need to study this area is not limited to the prevalence of these problems. It is also supported by the inadequacy of existing interventions to improve young people's mental health and increase their well-being [34]. For example, many intervention programs offer short-term effects but fail to provide long-term benefits [54]. Moreover, the majority of existing programs ignore cultural and contextual compatibility in meeting the needs of young people [5]. The development of more large-scale and effective intervention programs is a critical need to increase the well-being of young people.

In order to address the shortcomings of previous studies and respond to current needs, ACT (Acceptance and Commitment Therapy) based positive psychology interventions offer a promising approach to improve the mental health of young people [53]. Such interventions utilize evidence-based techniques to support individuals to cope with challenges and promote a meaningful life [49]. ACT-based approaches are seen as an effective way to increase individuals' subjective well-being by enabling them to develop positive thoughts and behaviors [58]. In this context, it is critical to develop more long-term, sustainable and effective intervention strategies to support young people's mental health and well-being.

### Why it is Important to Emphasize Well-Being in Youth

Positive psychology is a discipline that explores the processes that allow individuals to discover their potential and improve their quality of life [89]. This approach aims for individuals not only to be free from illness, but also to find satisfaction and meaning in life [91]. In the positive psychology perspective, well-being is addressed with two main concepts: subjective and psychological well-being. Subjective well-being is defined as individuals' satisfaction with their lives and the predominance of positive emotions and low levels of negative emotions [40], while psychological well-being is associated with individuals' capacity to achieve meaningful goals, develop positive relationships and utilize their strengths [85]. Seligman's PERMA model extends these approaches and defines five basic dimensions of well-being: positive emotions, engagement, relationships, meaning and accomplishment. This model explains the basic elements that increase individuals' life satisfaction and strengthen their mental health [91].

Youth is a period in which individuals form their identities, determine their personal values and develop goals for the future [3, 4]. During this period, individuals experience emotional, cognitive and social changes and use their internal and external resources to adapt to these changes [86, 87]. In this process, well-being allows individuals to cope with the stress and difficulties they face by increasing their resilience [13]. Young people with high well-being tend to be more successful in problem-solving skills, more effective in social relationships and have higher levels of life satisfaction [14]. These characteristics make it easier to cope with situations such as identity crises, academic difficulties and social adaptation problems experienced during youth [8]. Thus, it can be stated that a high level of well-being is a critical factor that protects and improves individuals' mental health. In particular, positive emotions facilitate coping with stress and increase resilience [17, 46]. For example, positive emotions enable individuals to think more creatively and flexibly by expanding their attention, which in turn improves problem-solving skills [47]. In addition, high life satisfaction strengthens individuals' resilience and reduces the risk of mental problems such as depression and anxiety [42, 67]. In this context, positive psychology interventions also contribute to the prevention of mental health problems by aiming to develop positive characteristics and strengths of individuals [90].

The effects of well-being on individuals are not independent of the cultural context. In collectivist cultures such as Türkiye, the impact of social relationships and community support on well-being is more evident [41, 100]. Furthermore, in Türkiye, religion and spiritual values play an important role in individuals' processes of finding meaning, and this is among the local factors that support well-being [2]. However, given the social, economic, and academic challenges that young people face, culturally adapted interventions are needed to enhance well-being during this period [14]. The dissemination of positive psychology interventions in the Turkish context can improve the quality of life of individuals by increasing their resilience and enable young people to contribute more effectively to society in the future.

### Positive Psychology Interventions

Positive psychology is an approach that aims to improve well-being and quality of life based on developing individuals' strengths and focusing on positive experiences [16, 89]. Interventions in this field support individuals to lead lives based on happiness, meaning and strengths [88]. Positive psychology interventions (PPIs) are scientifically validated methods designed to achieve goals such as promoting positive emotions, building positive relationships, and enhancing individual development [94]. The basic principles of these interventions are to provide scientifically based strategies that aim to increase individuals' well-being and strengthen resilience [91].

PPIs have been found effective in both increasing well-being and reducing negative symptoms such as depression, anxiety and stress [29]. For example, practices such as gratitude journaling increased life satisfaction [43], while interventions integrated with cognitive behavioral therapy reduced symptoms of anxiety and depression [30]. Multicomponent PPIs are known to have significant effects on well-being, depression and stress [54]. In addition, these effects of PPIs were found to be sustainable in follow-up periods [70]. PPIs with young people aim to provide strategies appropriate to the mental health and well-being needs of this age group. Young people are in the developmental processes of identity formation, deepening social relationships and determining their life goals [3, 4]. These processes, when supported by PPIs, lead to positive outcomes in areas such as life satisfaction, self-efficacy, and emotional resilience [92, 93]. Programs designed for youth can have long-term effects on academic achievement, social interactions, and emotional development. For example, positive psychology programs such as EmpowerU support young people to recognize their strengths, build emotional resilience, and develop positive relationships [14, 70].

Studies with young people show the effectiveness of interventions such as keeping a gratitude diary, identifying strengths, developing optimism and mindfulness-based techniques [104]. Gratitude journaling practices increased positive emotions and reduced the risk of depression [43]. Interventions focusing on strengths strengthened individuals' self-efficacy and life meaning [81]. Moreover, mindfulness-based interventions improved quality of life by supporting stress management and positive thinking skills [60]. By integrating these techniques, multicomponent programs offer holistic support to individuals' lives and provide effective strategies that they can easily apply in their daily lives [14]. These findings support the feasibility and impact of PPIs with young people, while demonstrating the potential to build on their strengths and positive emotional experiences.

### EmpowerU: ACT-Based Positive Psychology Intervention

ACT is an approach that aims to help individuals gain psychological flexibility. The basic principles of ACT are based on mindfulness, acceptance, values-aligned living and cognitive defusion processes [51]. Psychological flexibility refers to a capacity that enables individuals to exhibit effective and value-oriented behaviors while coping with difficulties [58]. This process aims to help individuals approach their thoughts and feelings openly, accept these experiences, and sustain their lives in a meaningful way. Mindfulness practices enable individuals to focus on the present moment and develop their emotional awareness [24].

When combined with the principles of positive psychology, ACT provides an effective framework for enhancing individuals' subjective well-being [19]. While positive psychology focuses on the development of positive emotions, meaningful life and individual strengths, ACT supports these processes with psychological flexibility and acceptance skills [59]. For example, mindfulness-based interventions mediate an increase in positive emotional experiences by helping individuals develop feelings of gratitude and find meaning in their lives [48]. The principle of value-based living allows individuals to define their self-worth and lead a life in accordance with these values [53]. Thus, individuals can not only manage their psychological difficulties but also develop sustainable resources that enhance their well-being.

Especially for young individuals, ACT can be an important tool for increasing self-awareness and developing psychological flexibility. It is known that young people under intense academic and social pressures need to develop psychological flexibility to cope with these pressures [27, 36]. ACT's mindfulness-focused practices support young people to cope with stress more effectively, increase their positive emotions and add meaning to their lives [52, 97]. Value identification processes and the development of strengths contribute to young people's academic success and increase their self-confidence in a social context [79, 81].

The impact of ACT on individuals is not only limited to individual well-being, but also enables the strengthening of social relationships [72, 80]. ACT, which has positive effects especially on empathy, emotional awareness and decision-making skills, contributes to the development of social bonds and effective use of interpersonal support mechanisms [53, 65]. The literature shows that ACT-based interventions reduce symptoms of depression, anxiety, and stress, as well as increase life satisfaction, positive emotions, and sense of self-efficacy [80, 84]. For example, ACT interventions including mindfulness practices have been proven to improve individuals' emotional regulation skills and reduce physical symptoms of stress [45].

Longitudinal studies show that the impact of ACT on individuals' well-being is sustainable not only during the intervention process but also in the long term [1, 50]. Research, especially with young individuals, reveals that ACT helps individuals to recognize their strengths, lead a value-oriented life, and develop positive social bonds [66]. Integrated with positive psychology, ACT aims to increase individuals' subjective well-being by enabling them to develop long-term behavioral changes [59]. These findings strongly suggest that ACT-based positive psychology interventions can offer an effective approach to support young individuals' mental health and general well-being.

### The Present Study

This study aims to examine the longitudinal effects of an ACT-based PPI on young people's mental health and well-being. Specifically, it evaluates the long-term effectiveness of a multi-component intervention designed to enhance subjective well-being, self-compassion, optimism, and resilience, while reducing pessimism, anxiety, depressive symptoms, and somatic complaints. Although ACT-based interventions have shown promise, research on their sustained impact—particularly in youth populations—remains limited in the broader literature [50, 78], and especially in the Turkish context [19, 23], where studies tend to focus on short-term outcomes. Addressing this gap, the present study provides both theoretical and practical contributions by evaluating an intervention model and offering a framework for long-term strategies that support youth mental health. Based on prior evidence, the following hypotheses were proposed: (*H*_*1*_) that ACT-based PPI would have a positive impact on youth well-being indicators (e.g., subjective well-being, self-compassion, optimism, resilience); (*H*_*2*_) that ACT-based PPI would have a negative impact on youth negative mental health outcomes (e.g., pessimism, anxiety, depression, somatization); and (*H*_*3*_) that the effects of ACT-based PPI would last post-intervention and throughout the follow-up period.

## Method

### Participants

The study was conducted at a state university located in the Mediterranean region of Türkiye. The study was announced in the Faculty of Education, Faculty of Economics and Administrative Sciences, and Faculty of Health Sciences of the university. 50 people applied to participate in the study voluntarily. Then, one-on-one interviews were conducted with 50 people, and they were informed about the study. 8 people who did not meet the study criteria (please see the procedure section for more information) were excluded and 42 people were randomly determined to be assigned to the experimental and control groups. 42 people were assigned to the experimental and control groups. The final groups (experimental = 20, control = 22) were formed. There were 12 women (60%) and 8 men (40%) between the ages of 19 and 23 in the experimental group (Mean age = 21.30, *SD* = 1.08). The control group included 16 women (72.70%) and 6 men (27.30%) between the ages of 20 and 23 (Mean age = 21.30, *SD* = 0.71).

### Measures

#### Subjective Well-Being

The Comprehensive Inventory of Thriving (CIT) is a self-report scale that aims to assess the psychological well-being of individuals consisting of 54 items [96]. In this inventory, there are 18 subscales consisting of 3 items each and the data obtained are associated with seven psychological constructs. Subjective well-being is one of these seven psychological constructs and is related to life satisfaction, positive emotions and negative emotions subscales. The scale items are in 5-point Likert scale (*1* = *strongly disagree* to *5* = *strongly agree*). Higher scores indicate higher subjective well-being. In this study, life satisfaction, positive emotions and negative emotions subscales (nine items in total) were used to indicate subjective well-being (e.g., *my life is going well*). This scale has adequate psychometric properties for the Turkish sample [10]. In the present study, the internal reliability estimates of the measures were adequate-to-strong (α range = 0.78 to 0.94).

#### Self-Compassion Scale

The Self-Compassion Scale-Short, which aims to measure individuals' level of self-compassion, consists of 12 items [82]. The scale is a 5-point Likert scale (*1* = *almost never* to *5* = *almost always*) and the higher the scores, the higher the level of self-compassion (e.g., *I try to see my failures as part of being human*). The scale has adequate psychometric properties for the Turkish sample [12, 15]. In the present study, the internal reliability estimates of the measure was strong (α range = 0.88 to 0.90).

#### Adult Resilience Measure (ARM)

ARM [83] is a 21-item self-report scale adapted into Turkish by Arslan [6]. The scale items are 5-point Likert-type (*1* = *does not describe me at all* to *5* = *describes me completely*). The scale consists of four dimensions (relational resources, individual resources, cultural and contextual resources, familial resources). A high score on the scale is associated with a high level of psychological resilience (e.g., *I am cooperative with the people around me*). The scale has adequate psychometric properties for the Turkish sample [6, 7]. In the present study, the internal reliability estimates of the measure was strong (α range = 0.81 to 0.90).

#### Optimism and Pessimism Measure (OPM-Short)

The Optimism–Pessimism Scale is a 12-item, two-dimensional (optimism, pessimism) self-report scale developed to assess the optimism and pessimism levels of young people and adults in Turkish culture [21]. The items are 5-point Likert-type (e.g., *I usually have a positive outlook on life; 1* = *strongly disagree* to *5* = *strongly agree*). Research has shown that the scale has strong internal reliability estimates [21]. In the present study, the internal reliability estimates of optimism and pessimism were strong (α range = 0.85 to 0.89).

#### The Brief Symptom Inventory (BSI- 18)

The BSI-18 is an 18-item self-report scale consisting of 3 dimensions (depression, anxiety and somatization) developed to assess psychological symptoms of adults [38]. The scale is a 5-point Likert scale and is scored from 0 to 4 (e.g., *feeling of weakness, powerlessness in some parts of the body; 0* = *not at all* to *4* = *very much*). The scale has strong internal reliability estimates in Turkish culture [22]. In the present study, the internal reliability estimates of optimism and pessimism were strong (α range = 0.82 to 0.92).

### Procedure and Intervention

This study followed a structured process for evaluating the effectiveness of the EmpowerU intervention with college students [11]. The EmpowerU intervention is designed to improve resilience and overall well-being and includes a wide range of skills and strategies, such as discovering and aligning with values, embracing acceptance, gaining a deeper understanding of thoughts and emotions, recognizing character strengths, building strong and healthy relationships, seeking meaning and purpose in life, exploring altruism and selflessness, shifting perspective to see challenges as opportunities for growth, and practicing emotional coping skills and positive habits to facilitate growth and well-being. EmpowerU consists of 8 to 12 sessions, each 90 to 120 min in length. It uses a structured curriculum that combines positive psychology, acceptance and commitment, and cognitive-behavioral techniques (see Table 1).

**Table 1:** The EmpowerU intervention

Topic	Description
Embracing Resilience	Introducing group members, agreeing group guidelines and understanding characteristics of resilience. Writing a self-story.
Discovering Values	Discovering what is most important to yourself. Discover and acknowledge your core beliefs and values that shape your behavior and influence emotional and behavioral reactions, helping you to align your actions with what truly matters.
Gain Perspective	Gaining a deeper understanding of one’s thoughts and emotions, allowing them to respond to life's ups and downs with greater clarity and purpose.
Acceptance	Embrace life as it unfolds, cultivating an open and non-judgmental stance that provides the clarity needed to make informed decisions and make peace with inner experiences.
Emotional Strength	Emphasize the significance of nurturing joy, gratitude, love, and hope. Engage in savoring life experiences across the past, present, and future. Encourage them to be compassionate towards themselves.
Character Strengths	Identify your top character strengths to boost resilience and well-being. Recognize and appreciate the strengths in others, utilizing these qualities to surmount challenges, live your values authentically, and enhance overall well-being.
Build Strong Relationships	Cultivate meaningful connections characterized by mutual respect, trust, and active engagement.
Meaning & Purpose	Focus on the pursuit of meaning and purpose in life by engaging in activities that align with your deepest values and aspirations.
The Power of Belief for the Future	Shift perspective to view challenges as opportunities for growth and transformation. Adversity is not something to be avoided but rather a chance to learn, adapt, and become more resilient.
Moving Forward	Practice new ways of relating to emotions and keep the developing coping skills and practicing positive habits to facilitate personal growth and well-being.

*Note.* The power of belief for the future and moving forward topic were practiced in the last session

**Fig. 1:**
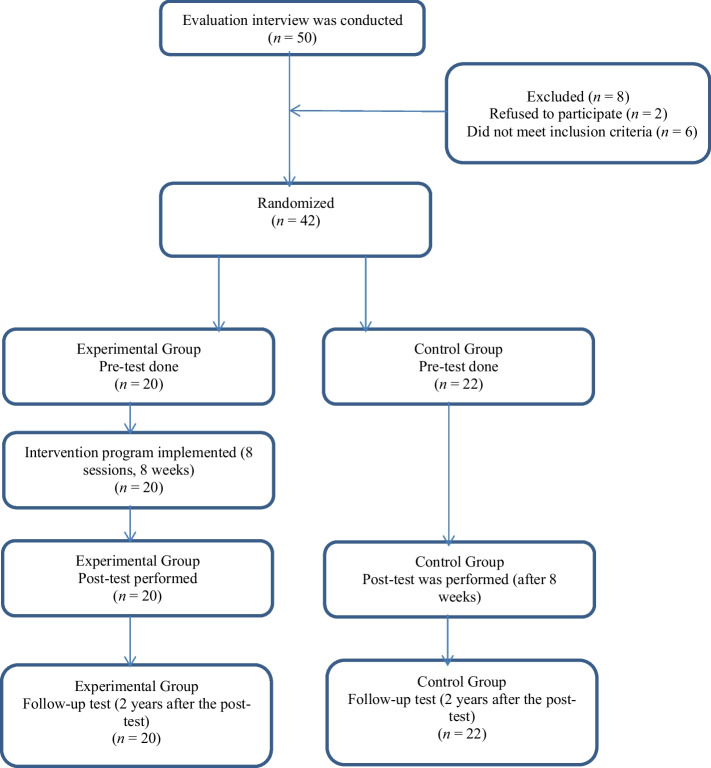
Flow chart illustrating the changes in participants throughout the process

Participants (*N* = 50) underwent an initial assessment interview (see Fig. 1). Eight participants were excluded: two declined to participate, and six did not meet the inclusion criteria, which included completing the pre-test and meeting the study's eligibility requirements regarding health status (e.g., no chronic health problems). The remaining 42 participants were randomly assigned to two groups through a lottery draw, ensuring that each participant had an equal chance of being placed in either the experimental or control group. They were informed that the study was conducted voluntarily and that they could withdraw from the study at any time. Participants were assigned to the experimental group (*n* = 22; however, two participants were unable to attend the weekly sessions, resulting in a final sample of 20) and the control group (*n* = 22). In the experimental group, the intervention program was implemented for 8 weeks (8 sessions). All sessions included at least one positive psychology concept, and each session (except the first session) followed the stages of summary of the previous week, review of homework assignments, discussion of the agenda, summary, determination of homework assignments, and receiving feedback. The intervention program included the themes of embracing resilience; accepting, gain perspective, discovering values, emotional strength, character strengths, meaning and purpose, building positive relationships, the power of belief in the future, and moving forward (see Table 1). No application was made to the control group. After 8 weeks (after the intervention program applied to the experimental group ended), a post-test was administered to all participants. The intervention program was conducted between September 19, 2022, and November 8, 2022. Following the end of the intervention program, students graduated from university, and on February 6, 2023, earthquakes of magnitude 7.7 and 7.6 occurred in Türkiye centered in Kahramanmaraş and Elbistan. More than fifty thousand citizens died in this earthquake and millions of people were affected. For this reason, the follow-up test could not be conducted in 2023. After the individuals returned to their daily routines, the participants were contacted, and it was learned that all of them were alive. A follow-up test was conducted for all participants in both groups two years after the post-test to evaluate the long-term effects of the intervention. This test was conducted by sending an online form to the individuals' phones. This structured approach ensured consistent assessment of both short-term and long-term outcomes while adhering to the study design.

### Data Analyses

We first examined descriptive statistics and assessed the assumptions of the analysis for pre-test, post-test, and follow-up (approximately two years later) scores on well-being (or positive psychological outcomes) indicators (e.g., subjective wellbeing, resilience, and self-compassion) and psychological health problems (or negative psychological outcomes, e.g., depressive symptoms, somatization, and pessimism). We examined the normality of the variables using skewness and kurtosis scores. ANOVAs (analyses of variance) were initially conducted to check for differences in pre-test scores between the experimental and control groups. Then, primary analyses were performed using analyses of covariance (ANCOVAs) to evaluate the effect of the ACT-based PPI on psychological health and well-being, while controlling for pre-test scores in both groups. ANCOVA is a robust and powerful technique for assessing group differences in randomized control designs, especially when random assignment is not possible or when there is a significant difference between the groups' pre-test scores [55, 95]. The effect size of the intervention was measured using partial eta squared (η_p_^2^), with the following conventional thresholds: 0.01 to 0.059 for small, 0.06 to 0.139 for medium, and 0.14 or higher for large effects. All data analyses were performed using SPSS v27 for Windows.

## Results

### Preliminary Analyses

Preliminary analyses demonstrated that all variables in the study had relatively normal distribution [35, 37]. Skewness and kurtosis scores were lower than |3| and ranged between −1.57 and 2.96. In the preliminary analyses of variance on group differences in pre-test scores for well-being and psychological health measures showed a few significant differences between experimental and control group, with small to medium effect sizes: subjective wellbeing, *F*(1, 41) = 1.04, *p* = 0.313, *η*^2^ = 0.03, self-compassion, *F*(1, 41) = 0.01, *p* = 0.965, *η*^2^ = 0.00, resilience, *F*(1, 41) = 0.70 *p* = 0.407, *η*^2^ = 0.02, optimism, *F*(1, 41) = 0.09, *p* = 0.764, *η*^2^ = 0.00, pessimism, *F*(1, 41) = 0.00, *p* = 0.990, *η*^2^ = 0.16, depression,* F*(1, 41) = 5.25, *p* = 0.027, *η*^2^ = 0.12, anxiety, *F*(1, 41) = 4.08, *p* = 0.050, *η*^2^ = 0.09, and somatization, *F*(1, 41) = 2.78, *p* = 0.104, *η*^2^ = 0.07 (see Table 2). Further analyses were conducted to evaluate the assumptions required for ANCOVA, including the homogeneity of variance, the normal distribution of residuals, and the homogeneity of regression slopes across groups. The results of these evaluations confirmed that the data met the necessary criteria, indicating that ANCOVA was an appropriate and robust statistical approach to assess the effectiveness of the ACT-based PPI.

**Table 2:** ANOVAs and ANCOVAs results

	Pre-test					
	Experimental		Control			
	M	SD	M	SD	*p*	*η*^*2*^
Subjective Wellbeing	12.75	5.69	11.04	5.12	0.313	0.03
Self-compassion	40.70	7.86	40.59	8.09	0.965	0.00
Resilience	80.00	10.62	82.59	9.41	0.407	0.02
Optimism	22.25	3.95	21.86	4.30	0.764	0.00
Pessimism	12.70	5.61	12.68	4.15	0.990	0.00
Depressive Symptoms	6.50	4.56	9.68	4.43	0.027	0.12
Anxiety	5.65	4.12	8.81	5.80	0.050	0.09
Somatic Symptoms	4.65	4.46	7.36	5.90	0.104	0.07

	Post-test					
	Experimental		Control			
	M	SD	M	SD	*p*	*η*^*2*^
Subjective Wellbeing	16.20	5.35	11.27	6.34	0.018	0.14
Self-compassion	46.15	8.24	40.18	7.40	0.005	0.19
Resilience	83.75	8.38	82.41	11.74	0.142	0.05
Optimism	24.70	3.48	22.09	4.77	0.033	0.11
Pessimism	9.80	3.75	14.68	6.66	0.002	0.22
Depressive Symptoms	4.30	2.49	9.18	5.16	0.007	0.17
Anxiety	3.50	1.93	8.82	6.04	0.003	0.20
Somatic Symptoms	3.35	2.89	7.05	5.86	0.039	0.11

	Follow-up (two years later)					
	Experimental		Control			
	M	SD	M	SD	*p*	*η*^*2*^
Subjective Wellbeing	16.15	4.89	11.00	5.51	0.004	0.19
Self-compassion	42.55	6.80	32.73	5.68	< 0.001	0.43
Resilience	84.25	8.87	73.50	5.92	< 0.001	0.36
Optimism	24.65	3.38	21.50	3.49	0.006	0.18
Pessimism	10.35	4.56	12.82	3.16	0.048	0.10
Depressive Symptoms	4.35	1.90	8.86	3.14	< 0.001	0.42
Anxiety	4.75	2.51	7.14	5.30	0.123	0.06
Somatic Symptoms	2.75	2.63	5.41	4.89	0.053	0.09

### Primary Analyses

Prior to conducting the ANCOVAs, the homogeneity assumption of the regression slopes was tested for each dependent variable, and no significant interaction terms were found, meeting the necessary assumptions for ANCOVA. Results of the analyses showed several significant main effects of the intervention on positive and negative psychological outcomes, with varying effect sizes and sustainability of changes at follow-up. Descriptive statistics and ANCOVA results for each dependent variable are presented in Table 2.

With regard to positive psychological outcomes, participants in the experimental group reported significantly higher subjective well-being (*F*[1, 41] = 6.09, *p* = 0.018, *η*_*p*_^2^ = 0.14) and self-compassion (*F*[1, 41] = 9.05, *p* = 0.005, *η*_*p*_^2^ = 0.19) scores than those in the control group at post-test. This difference increased at follow-up, with the experimental group continuing to report significantly higher well-being (*F*[1, 41] = 9.32, *p* = 0.004, *η*_*p*_^2^ = 0.19) and self-compassion (*F*[1, 41] = 29.09, *p* < 0.001, *η*_*p*_^2^ = 0.43) than the control group, with large effect sizes. In addition, although there were no significant differences between the experimental and control groups at post-test (*F*[1, 41] = 2.24, *p* = 0.142, *η*_*p*_^2^ = 0.05), the experimental group reported significantly higher levels of resilience than the control group at follow-up (*F*[1, 41] = 1.04, *p* < 0.001, *η*_*p*_^2^ = 0.36), and the effect size was large. Similarly, the experimental group reported significantly higher optimism scores (*F*[1, 41] = 4.88,* p* = 0.033, *η*_*p*_^2^ = 0.11) than the control group at posttest, with a moderate effect size. This effect was maintained at follow-up (*F*[1, 41] = 8.52, *p* = 0.006, *η*_*p*_^2^ = 0.18), with the experimental group maintaining higher levels of optimism than the control group, with a large effect size.

**Fig. 2:**
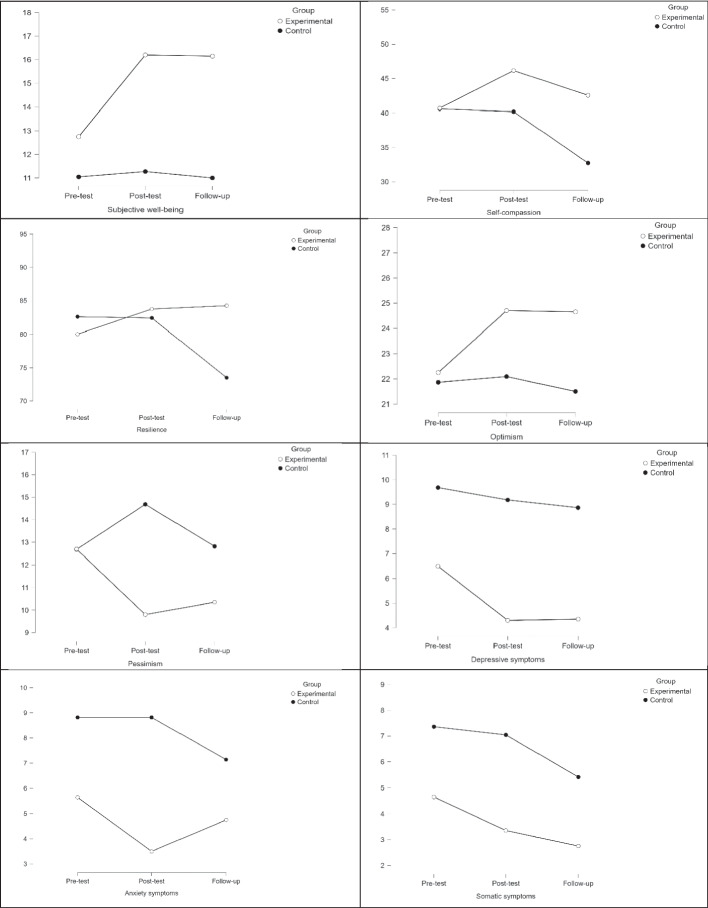
Effects of ACT-based positive psychology intervention on the variables of the study across three times (pre-test, before intervention; post-test, after intervention; follow-up, 2 years after intervention)

Regarding negative psychological outcomes, a significant decrease in pessimism was observed in the experimental group at posttest compared to the control group (*F*[1, 41] = 10.90, *p* = 0.002, *η*_*p*_^2^ = 0.22), with a large effect size. At follow-up, the experimental group continued to have lower levels of pessimism than the control group, with moderate effect size (*F*[1, 41] = 4.18, *p* = 0.048, *η*_*p*_^2^ = 0.10). For psychological distress indicators, participants in the experimental group reported lower scores for depressive symptoms (*F*[1, 41] = 8.18, *p* = 0.007, *η*_*p*_^2^ = 0.17), anxiety (*F*[1, 41] = 9.84, *p* = 0.003, *η*_*p*_^2^ = 0.20), and somatic symptoms (*F*[1, 41] = 4.57, *p* = 0.039, *η*_*p*_^2^ = 0.11) at posttest compared to those in the control group. These changes were maintained at follow-up for depressive (*F*[1, 41] = 27.65, *p* < 0.001, *η*_*p*_^2^ = 0.42) and somatic symptoms (*F*[1, 41] = 3.99, *p* = 0.053, *η*_*p*_^2^ = 0.09), with the experimental group continuing to have lower levels of these symptoms than the control group, with moderate and large effect sizes. However, no significant differences were observed at follow-up for anxiety symptoms (*F*[1, 41] = 2.49, *p* = 0.123, *η*_*p*_^2^ = 0.06). Overall, the ACT-based PPI showed moderate to large effects on both improving positive psychological outcomes (e.g., subjective well-being, self-compassion, resilience) and reducing negative psychological health symptoms (e.g., depressive symptoms, pessimism), with many of the effects persisting two years after the intervention. The effects of ACT-based PPI on the study variables across the three time points are presented in Fig. 2.

## Discussion

The results of this study reveal that the ACT-based PPI had significant effects on both positive and negative psychological outcomes for youth. In terms of positive psychological outcomes, participants in the experimental group showed a significant increase in subjective well-being and self-compassion scores at post-test compared to the control group. Importantly, these effects were even more pronounced during the follow-up test and were maintained as long-term gains. It is consistent with previous research that such interventions on young people strengthen their positive psychological characteristics and increase their emotional resilience at a critical period of their lives [23, 29, 92].

The significant improvement in self-compassion observed in this study suggests that young people developed a kinder and more forgiving attitude toward themselves when facing emotional difficulties. Self-compassion is known to support mental well-being by reducing self-criticism and fostering emotional resilience, a finding consistent with previous research [57, 63, 74, 77]. Within the Turkish cultural context, where family and community ties are highly valued, such gains may have been further reinforced through social support mechanisms that facilitate emotional growth [56]. This aligns with studies showing that positive shifts in self-perception are often reflected in improved academic and social functioning [63, 75]. However, it is also important to consider that in collectivist cultures like Türkiye, social norms that emphasize conformity and relational harmony may at times discourage overt emotional expression or self-disclosure. Such dynamics could influence how self-compassion is internalized or enacted, potentially moderating the full impact of interventions targeting this construct.

In terms of subjective well-being, the fact that young people showed that they were satisfied with their lives and had positive emotional experiences reveals that the intervention made a significant contribution to the quality of life of individuals. This is in line with the literature indicating that subjective well-being has a critical importance in the developmental stages of young people [103]. These gains in subjective well-being may help young people develop a more positive perspective in the face of stressful life events [18]. In addition, this may lead to a more hopeful attitude towards the future and a stronger construction of personal meaning [62, 91].

These results emphasize that ACT-based PPIs can have a strong impact on young people and that these effects can contribute to a healthier social and psychological development of young people. As a matter of fact, in previous studies, increasing self-compassion and subjective well-being in youth stands out as critical factors that strengthen individuals' capacity to cope with stress and uncertainty [28, 69, 76]. In particular, the follow-up test results of this study show that young people are able to maintain and even improve their gains even during stressful periods such as post-graduation work life. This suggests that ACT-based PPIs can provide not only short-term but also long-term benefits.

Increases in resilience and optimism suggest that young people have become more resilient to challenges and can look to the future with more hope. However, the fact that there was no significant difference in resilience at post-test suggests that the development of this trait requires a longer-term process. This difference emerged in the follow-up test shows that the young people were able to maintain the gains they had made even in stressful processes such as post-graduation work life and even carry these gains further. This supports that resilience is a capacity that develops over time and that the effects of interventions made during youth may emerge as individuals transition to different stages of life [71]. The observed increases in optimism may have supported young people's capacity to evaluate life events from a more positive perspective after the intervention. In particular, these results are in line with the literature that optimism strengthens young people's coping mechanisms with stress and increases their overall life satisfaction [106, 107]. For young people, optimism is not only a characteristic that supports positive emotions, but also a resource that increases resilience in the face of challenging life events [39]. The sustainability of optimism levels in follow-up test results suggests that young people's capacity to develop positive expectations for the future can provide long-term benefits.

In the context of Turkish culture, optimism supported by strong social solidarity and interpersonal support systems may play a significant role in enhancing young people’s life satisfaction. In a society where collectivist values prevail, access to community-based support may help individuals manage stress and uncertainty more effectively, particularly during transitional periods such as post-graduation [56, 100]. For instance, young people entering the workforce may be better equipped to navigate challenges when they possess a strong sense of optimism. Interventions that foster resilience and optimism during this critical developmental stage can facilitate personal and social adaptation while promoting a more hopeful orientation toward the future. Cultivating these traits in youth not only enhances their ability to cope with present difficulties but also prepares them to confront future adversities with greater psychological resources [71, 102]. These findings suggest that resilience- and optimism-based interventions may be particularly impactful within cultural frameworks that emphasize social cohesion. However, collectivist norms that prioritize harmony and interdependence may, at times, constrain individual emotional autonomy or discourage the pursuit of personal aspirations, potentially influencing how optimism is internalized and maintained over time. Therefore, cultural values should be carefully considered in the interpretation of outcomes and in the design of future interventions.

In terms of negative psychological outcomes, young participants in the experimental group showed significant reductions in pessimism, anxiety, depressive symptoms and somatic symptoms compared to the control group. Youth is a stage of development when individuals are most susceptible to emotional changes and stressors [3, 4]. This finding suggests that the intervention may help young people to break negative thought cycles by increasing their mental flexibility and thus help them cope with negative emotions in a healthier way. In particular, the improvement in depressive and somatic symptoms was maintained during follow-up testing, supporting the long-term clinical benefits of the intervention and its potential to create lasting positive effects in young people's lives.

The reduction in pessimism observed in this study suggests that young people developed a greater capacity to adopt a more positive outlook, indicating the effectiveness of the intervention in challenging and transforming negative thought patterns. As pessimism is known to adversely affect psychological well-being by exacerbating stress and anxiety levels [20], its sustained decline throughout the follow-up period highlights the long-term benefits of the ACT-based intervention. Within the Turkish cultural context, strong social support networks and community-oriented values may have played a facilitative role in weakening persistent negative cognitions such as pessimism [56]. These results align with previous findings demonstrating the efficacy of PPIs in reducing maladaptive thinking patterns [25, 64]. However, it is also important to consider that cultural norms—while protective in many ways—may sometimes limit perceived personal autonomy, potentially reinforcing pessimistic outlooks when individuals feel constrained by social or familial expectations.

The reduction in depressive symptoms suggests that young people's capacity to evaluate life events in a more positive way has improved and that this intervention offers an effective support mechanism. Previous research has confirmed that positive psychological traits such as self-compassion and subjective well-being play a critical role in reducing depressive symptoms by enabling individuals to develop a more compassionate, understanding and supportive attitude towards themselves [44, 68]. The present study shows that these characteristics are effective on Turkish youth and that these characteristics provide a strong supportive mechanism in the management of depression. Moreover, the fact that these effects were maintained during the two-year follow-up period emphasizes the potential of the intervention to provide a sustainable improvement.

It is likely that the observed decrease in somatic symptoms is associated with the development of young people's coping skills for managing stress. Previous research has shown that stress often manifests as physical symptoms in youth, particularly under conditions of psychological pressure [32, 33]. Within this framework, the reduction in somatic symptoms may reflect the intervention’s effectiveness in enhancing stress regulation. Components such as awareness, acceptance, identification of personal strengths, emotional recognition, and helping behaviors may have contributed significantly to this outcome by promoting adaptive coping mechanisms. The maintenance of these gains during the follow-up period further supports the long-term impact of the intervention. In the context of Turkish culture, where community support is a key protective factor in managing stress, these findings align well with the broader cultural environment [56, 98]. However, it should also be noted that in some segments of Turkish society, mental health stigma may discourage open emotional expression, leading individuals to somatize psychological distress. This cultural dynamic may not only shape how stress is experienced but also influence how it is reported and should therefore be taken into account when interpreting the results.

When the results obtained in terms of anxiety symptoms are examined, although a significant decrease was observed in the experimental group in the post-intervention period, this difference became insignificant during the follow-up test. This result suggests that anxiety is more sensitive to long-term stressors and uncertainties. The difficulties faced by young people in the post-graduation period, such as transition to work life, career uncertainties and social adaptation, may have been effective in the persistence of anxiety symptoms [99]. This is consistent with research indicating that anxiety may be shaped by an individual's sensitivity to perceived threats [31]. However, there is clearly a need for more specific interventions targeting the long-term effects of anxiety.

The finding that some effects (e.g., optimism) were sustained in the long term while others (e.g., anxiety) diminished suggests that the durability of psychological outcomes may depend on the nature of the construct. Optimism, as a relatively stable cognitive-affective orientation, may be more resistant to external fluctuations and more easily reinforced through everyday experiences. In contrast, anxiety symptoms are often more reactive to environmental stressors and transitions, such as post-graduation uncertainties, career stress, and economic instability—conditions particularly salient in the follow-up period. This difference highlights the need for booster sessions or continuous support mechanisms for maintaining improvements in more situationally sensitive symptoms. Taken together, the results of this study suggest that ACT-based PPIs are effective in reducing pessimism, anxiety, depression, and somatic symptoms and in enhancing young people's subjective well-being, self-compassion, optimism, and resilience. While the overall effects were robust, the persistence of certain symptoms—particularly anxiety—indicates that such outcomes may be more closely linked to long-term stressors and thus may require more targeted and sustained interventions. These findings are consistent with prior research demonstrating the beneficial impact of positive psychology interventions on youth mental health and underscore their potential as valuable tools for promoting psychological well-being. In the Turkish cultural context, community-oriented social support systems may have played a reinforcing role in amplifying the effectiveness of the intervention. At the same time, cultural norms that emphasize collective harmony over individual emotional expression may shape how participants internalize or report the benefits of such programs. These cultural dynamics should be taken into consideration when interpreting the outcomes and designing future interventions.

### Implications and Limitations

While the results of this study offer important theoretical and practical implications, they also have some limitations. First, the fact that the sample group consisted only of university students limits the generalizability of the findings to the general population, even though they focus on the young population. This raises questions about how the effects of the intervention may vary across different age groups or socio-demographic characteristics. In particular, it is unclear whether the effects would be similar for individuals with longer work experience or living in different cultural contexts. Second, the lack of random assignment to the intervention and control groups may have partially affected the internal validity of the study. Although this limitation was attempted to be controlled by ANCOVA, it is recommended that a more rigorous design be used in the future. In addition, the fact that the participants had started their professional life after graduation during the follow-up test is a factor that should be taken into account in the interpretation of the results. This may have increased the effects of stress and uncertainties in the post-graduation period, especially on anxiety symptoms. Third, as all variables were measured through self-report instruments, the findings may be subject to biases such as social desirability or inaccurate self-perceptions. These inherent limitations of self-report methods should be considered when interpreting the results. Another limitation of the study is that it focused only on specific positive and negative psychological indicators. A broader assessment is needed to understand how other important psychological characteristics such as hope, posttraumatic growth and stress are affected by the intervention.

Despite these limitations, the results of the study offer valuable implications from both theoretical and practical perspectives. The ACT-based PPI significantly contributed to the development of positive psychological traits such as subjective well-being, self-compassion, resilience and optimism by increasing young people's mindfulness and psychological flexibility. The long-term maintenance of reductions in depressive symptoms, pessimism and somatic symptoms demonstrates the potential of this intervention to improve young people's quality of life. In particular, the maintenance of gains in follow-up test results after two years supports the sustained effects of the intervention and demonstrates its long-term benefits.

In the context of Turkish culture, it is seen that community-oriented social support systems contribute to individuals' development of characteristics such as resilience and optimism. This suggests that the effects of the intervention are in line with cultural norms and that individuals can further reinforce these gains in their social context. Strengthening self-compassion not only increased young people's capacity to cope with inner criticism, but also supported their processes of seeking support and developing positive relationships in the social context. Gains in optimism enabled young people to look to the future with more hope, while improvements in resilience enabled them to become more resilient to stressful life events.

These results suggest that ACT-based PPIs should be disseminated especially in educational institutions. For young people, such as university students, who frequently encounter stress and uncertainty, such interventions can make positive contributions to both their academic and post-graduation lives. In addition, it is thought that such programs can be supportive in the career transition and adaptation processes of young people to work life. In the future, the generalizability and long-term effects of such interventions should be evaluated more comprehensively with future studies in different age groups and cultural contexts. It is recommended that such interventions be implemented on a larger scale as an effective tool to improve the quality of life of individuals.

## Data Availability

The data supporting the findings of this study are available from the corresponding author upon reasonable request.
